# Supplementary material for: Prevalence and predictors of depression, anxiety, and stress among adults in Ghana: A community-based cross-sectional study
Source: PLoS One. 2021 Oct 8;16(10):e0258105. doi: 10.1371/journal.pone.0258105 (PMC8500438; doi:10.1371/journal.pone.0258105)
Supplement: S2 Table — (DOCX) [file pone.0258105.s002.docx]

**Table S2. ﻿ Study variables**

| **Variables** | **Question** | **Response options and recoding** |
| --- | --- | --- |
| **Outcome variables** | | |
| **The DASS-21 scale:** All items on the scale were assessed as;  1 “Did not apply to me at all”  2 “Applied to me to some degree”  3 “Applied to me to a considerable degree”  4 “Applied to me a very much or most of the time”  **Coded as**  **0 “Did not apply to me at all”**  **1 “Applied to me to some degree”**  **2 “Applied to me to a considerable degree”**  **3 “Applied to me a very much or most of the time”** | | |
| Depression | I couldn't seem to experience any positive to do things |  |
|  | I found it difficult to work up the initiative to do things |  |
|  | I felt that U had nothing to look forward to |  |
|  | I felt down-hearted and blud |  |
|  | I was unable to become enthusiastic about anything |  |
|  | I felt I wasn't worth much as a person |  |
|  | I felt that life was meaningless |  |
|  |  |  |
| Stress | I found it hard to wind down |  |
|  | I tended to over-reach to situations |  |
|  | I felt that I was using a lot of nervous energy |  |
|  | I found myself getting agitated |  |
|  | I found it difficult to relax |  |
|  | I was intolerant of anything that kept me from getting on with what I was doing |  |
|  | I felt that I was rather touchy |  |
|  |  |  |
| Anxiety | I was aware of dryness of my mouth |  |
|  | I experienced breathing difficulty (e.g. excessively rapid breathing, breathlessness in the absence if physical exertion) |  |
|  | I experienced trembling (e.g. in the hands) |  |
|  | I was warried about situations in which I might panic and make a fool of myself |  |
|  | I felt I was close to panic |  |
|  | I was aware of the action of my heart in the absence of physical exertion (e.g. sense of heart rate increase, heart missing a beat) |  |
|  | I felt scared without any good reason |  |
|  | | |
| **Explanatory variables** | | |
| Study District | District | **Responses provided by data collectors**  Ketu South  Ho West  Hohoe  Nkwanta South |
| Age | How old are you | Open question  **(coded as 18-29=1, 30-39=2, 40-49=3, 50-59=4, 60+=5)** |
| Sex | Sex | 1=male, 2=female |
| Ethnicity | What is your Ethnicity? | 1=Ewe, 2=Akan, 3=Guan, 4= Ga/Dangme, 5=Mole-Dagbani, 6=other (Specify)  **(coded as 1=Ewe, 2=Akan, 3=Guan, 4= Ga/Dangme, 5=Mole-Dagbani)** |
| Religion | Religion | 1=Christianity, 2=Islam, 3= African Traditional  **(coded as 1=Christianity, 2=Muslim, 3= Traditionalist)** |
| Educational status | Educational status | 1=None, 2=Primary, 3=JHS, 4=SHS, 5=Tertiary |
| Occupation | What is your main occupation? | Open ended question  **(Coded as 1=Unemployed, 2=Government employed, 3=Self-employed)** |
| Income | What is your average monthly income? | **(Coded as 1=<500, 2=500-999, 3=≥1000)** |
| Hypertensive status | Systolic measurement  Diastolic measurement | **Normal:** (Systolic BP <120 and Diastolic BP <80 mmHg)  **Hypertensive:** Pre-hypertension (Systolic BP = 120-139 and/or Diastolic BP = 80-89 mmHg); Hypertension- Stage I hypertension (Systolic BP = 140-159 and/or Diastolic BP = 90-99 mmHg) and Stage II hypertension (Systolic BP > 160 and/or Diastolic BP > 100 mmHg). |
| Current smoking status | Do you currently smoke? | 0=No, 1= Yes  **(coded as 0= Non-current smoker, 1= current smoker)** |
| Current alcohol intake status | Do you currently drink? | 0=No, 1= Yes  **(coded as 0= Non-current smoker, 1= current smoker)** |
